# A Rare Case of Neuroinvasive West Nile Virus in Florida Presenting As Guillain Barré Syndrome

**DOI:** 10.7759/cureus.33138

**Published:** 2022-12-30

**Authors:** Monica Sciturro, Sarah Rehl, Jessica Klingensmith

**Affiliations:** 1 Medicine, Nova Southeastern University Dr. Kiran C. Patel College Of Osteopathic Medicine, Clearwater, USA; 2 Infectious Disease, Nova Southeastern University Dr. Kiran C. Patel College Of Osteopathic Medicine, Davie, USA; 3 Family Medicine, HCA Florida St. Petersburg Hospital, St. Petersburg, USA

**Keywords:** mosquito vector, zoonosis and public health, west nile, neuroinvasive west nile virus, guillain barre’s syndrome (gbs)

## Abstract

West Nile virus (WNV) is a leading cause of mosquito-borne illness in the continental United States. There are no vaccines to prevent or treat WNV, the mainstay of treatment is supportive care with rehydration, pain control, and possible antiemetic therapy. WNV is often asymptomatic but can rarely progress to a neuroinvasive disease, depicted by meningitis, encephalitis, and acute flaccid paralysis. This case report depicts a 64-year-old male who developed a rare neuroinvasive WNV in Florida. The patient was hospitalized for bilateral upper and lower extremity weakness, numbness, and tingling. CSF findings on admission were remarkable for albuminocytologic dissociation, suggesting that the patient was possibly suffering from isolated Guillain Barre Syndrome (GBS). The patient was treated with IVIG and plasmapheresis with no improvement in symptoms and later tested positive for WNV on day 22 of admission. This case highlights the variability in WNV presentation and CSF findings, highlighting the need for increased suspicion when patients present with findings consistent with GBS in the late summer months.

## Introduction

West Nile virus (WNV), a Japanese encephalitis virus antigenic complex member, can be transmitted by mosquitoes or rarely via blood transfusion or organ transplantation. The incubation period for WNV disease is typically 2 to 6 days but ranges from 2 to 14 days and can be several weeks in immunocompromised people. About 1 in 5 people who are infected develop a fever and other symptoms, while only 1 out of 150 to 250 develop neuroinvasive disease [1]. The neuroinvasive disease presents as fever in conjunction with meningitis, encephalitis, flaccid paralysis, rash, or a mixed pattern of illness [2]. Since WNV emerged in the United States in 1999, 22,999 neuroinvasive human disease cases have been reported through 2017 [3]. Here we discuss a neuroinvasive WNV that occurred in Florida in the summer of 2021 and was masked as Guillain Barre Syndrome (GBS).

GBS is suspected when patients develop ascending paralysis, often following an acute viral infection. After the first signs and symptoms, the condition tends to progressively worsen for about two weeks, peak within four weeks, and then recovery lasts roughly 6-12 months. Treatment for both WNV neuroinvasive disease and GBS is mainly supportive with IVIG used in adjunct; however, IVIG and corticosteroids have only shown intermittent success for WNV neuroinvasive disease. A thorough literature review shows few publications of signs and symptoms consistent with a variant for Guillain Barre as the presentation for WNV [4]. This case report is of particular importance as it depicts a different incidence of WNV presenting as Guillain Barre Syndrome, only this patient did not recover.

## Case presentation

**Figure 1:**
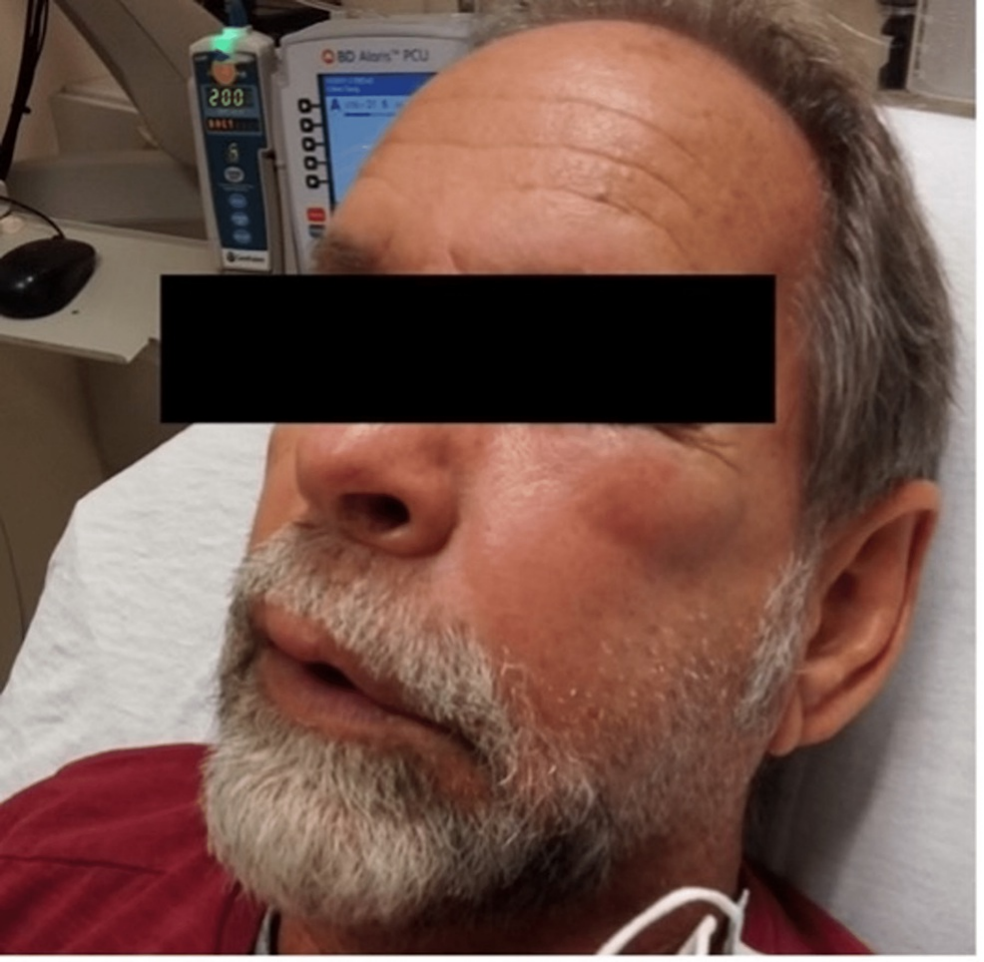
Patient’s preseptal cellulitis on left cheek 12 days before admission

**Figure 2:**
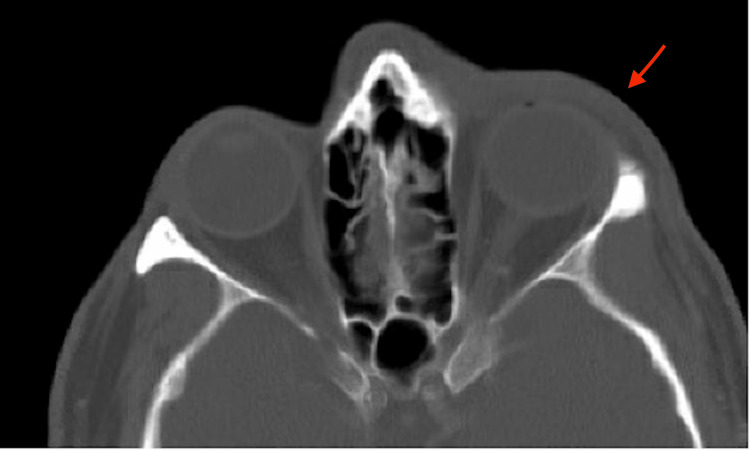
CT scan of the face with contrast indicates left periorbital preseptal cellulitis (red arrow) and left cheek cellulitis

A 64-year-old Caucasian male presented to the ED with generalized bilateral upper and lower extremity weakness, numbness, and tingling. His symptoms began 2 days prior with bilateral hand numbness and tingling after he finished trimming hedges outside in the heat. The symptoms progressed to bilateral upper and lower extremity weakness. He noted that his extremities felt “rubbery and heavy.” The patient also indicated new onset symptoms of difficulty lifting his arms, progressive inability to walk without assistance, decreased grip strength, difficulty swallowing, and crampy abdominal pain with constipation for the past 2 days. Vitals on admission were stable (Table 1). He was seen 12 days before this admission for left periorbital preseptal cellulitis and cellulitis of the cheek, confirmed by a CT scan of the face with contrast (Figures 1, 2). The patient was treated with oral Augmentin, Bactrim DS, and one IV course of Vancomycin and Unasyn. Bloodwork at that time was unremarkable. His past medical history was significant for asthma, diverticulitis, and nephrolithiasis. He denied any history of stroke or neuromuscular disorders, and his past surgical history consisted of left ear surgery for a ruptured tympanic membrane greater than 10 years ago. As for social history, the patient denied past or current tobacco, alcohol, or recreational drug use. He also denied any recent travel history.

**Table 1:** Patient vitals on admission

Variables	Result
Temperature	36.7 ℃ (98.06 ℉)
Blood Pressure	160/95 mmHg
Pulse	69 beats per minute
Respirations	24 breaths per minute
O_2_ saturation	97% on room air

**Figure 3:**
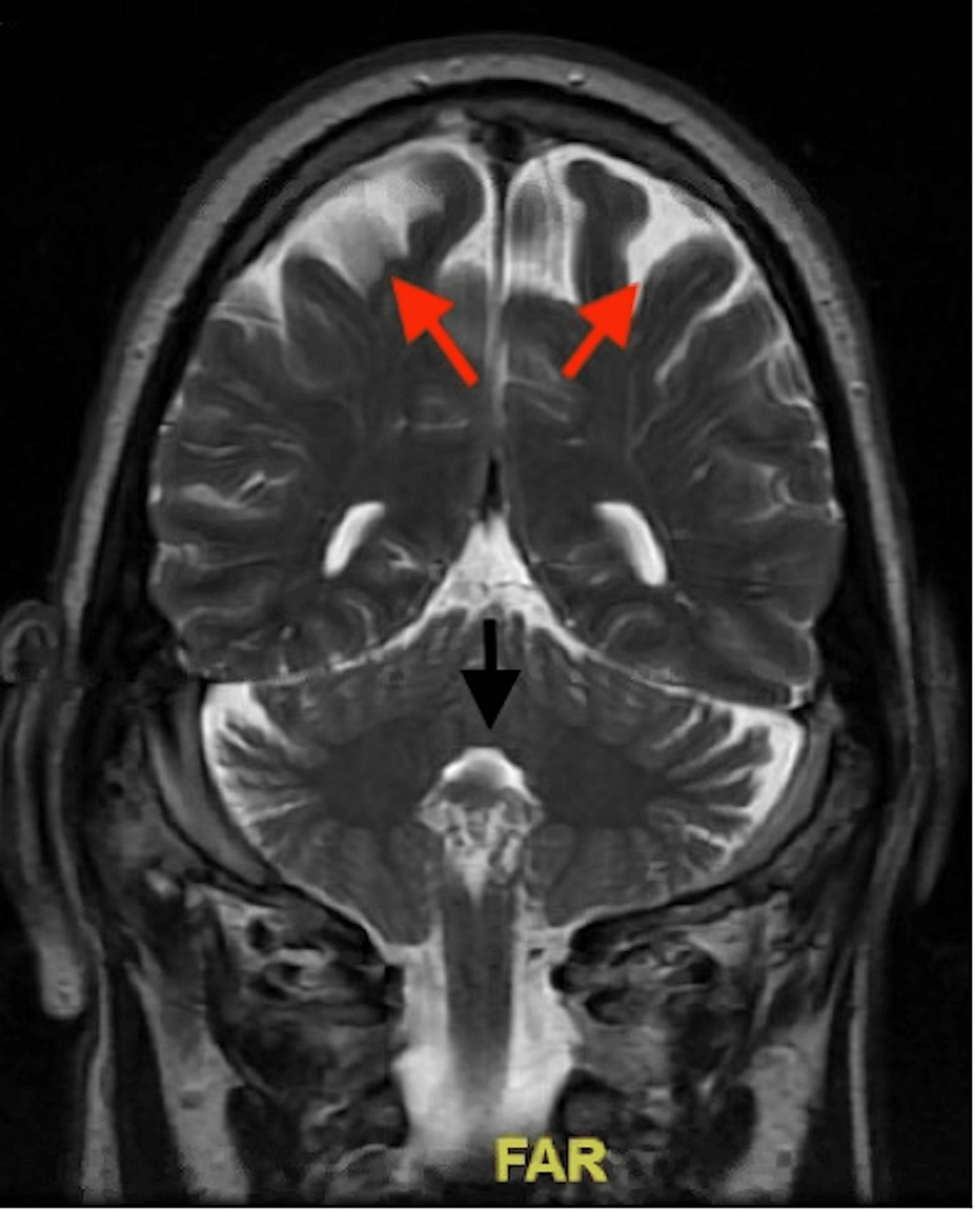
MRI brain with contrast indicates scattered foci of abnormally increased signal within the white matter of the cerebral hemispheres (red arrows) and pons (black arrow), most likely representing small vessel microvascular atheromatous change.

His physical exam was significant for bilateral lower extremity weakness 2/5, bilateral upper extremity weakness 4/5, with a sensory deficit in bilateral upper and lower extremities and abnormal reflexes. Initial labs were remarkable for elevated creatine kinase (CK) and creatine kinase-myoglobin binding (CK-MB) of 118 and leukocytosis of 12.02. MRI of the brain with and without contrast showed scattered foci of abnormally increased signal within the white matter of the cerebral hemispheres and pons, most likely representing small vessel microvascular atheromatous change (Figure 3).

The patient was admitted to the hospital ICU with an initial diagnosis of bilateral extremity weakness and neuropathy after a stroke alert was called for symptoms of worsening weakness. He was unable to hold his legs or arms up against gravity, unable to walk, had minimal grip strength, and diminished to absent deep tendon reflexes. He was subsequently intubated due to progressive weakness and respiratory failure. CSF studies showed albuminocytologic dissociation, suggestive of Guillain-Barre syndrome. The patient was initially treated with a 5-day course of IVIG with failure to respond. He then underwent 6 treatments of plasmapheresis (PLEX), also with no response.

Electroencephalography (EEG) revealed toxic metabolic encephalopathy; however, images of these EEG results are unavailable. Repeat CSF studies confirmed albuminocytologic dissociation with increased total protein and West Nile Virus IgG positive, IgM negative. The suspected etiology was possible facial cellulitis secondary to a mosquito bite on prior admission. Electromyogram and nerve conduction studies were consistent with atypical Guillain Barre with an Acute Motor Sensory Axonal Neuropathy (AMSAN) classification.

During his hospital stay, the patient received a tracheostomy and PEG tube. He completed treatment for sepsis due to West Nile encephalitis and hospital-acquired pneumonia with meropenem and micafungin. He was noted to have periods of altered mentation, and a repeat EEG showed moderate encephalopathy. Unfortunately, this patient underwent brief cardiac arrest before transferring to a long-term acute care hospital, after which he quickly deteriorated and was terminally extubated.

## Discussion

WNV can progress to neuroinvasive disease in less than 1% of patients and may present with acute flaccid paralysis without viral prodrome as it did in this patient. This can further progress to respiratory paralysis requiring mechanical ventilation with isolated limb paresis or paralysis. An extremely rare sequela is WNV-associated Guillain-Barré syndrome, which can be confirmed with CSF findings, EMG, and nerve conduction studies. In this case, the initial lumbar puncture and nerve studies were positive for albuminocytologic dissociation and axonal neuronal neuropathy, raising the suspicion solely for isolated Guillain Barre Syndrome. This emphasizes the need for a high clinical suspicion for vector-borne viral illnesses in tropical climates and the importance of a more in-depth initial viral evaluation when patient presentation resembles Guillain Barré Syndrome.

While the treatment options for GBS and WNV are similar, IVIG is significantly more effective in treating GBS than it has been in WN virus neuroinvasive disease. Corticosteroids have also been shown to be effective for neuroinvasive disease on a case-by-case basis, but antivirals like ribavirin and acyclovir lack efficacy. Without successful treatment for WNV neuroinvasive disease, the prognosis continues to be poor, with age being the most important predictor of risk. Furthermore, patients who recover can continue to experience incomplete recovery of limb strength and residual deficits [2]. One study found that 40% of individuals continue to experience symptoms 8 years after infection [5]. More research is needed to determine possible pharmaceutical or therapeutic treatment methods to eliminate this morbidity and mortality, seen first-hand in this case presentation.

Lastly, awareness and prevention of WNV have significantly progressed over the past 30 years. However, an increased need for improved vector surveillance remains as the infection continues to affect individuals in the United States, with positive cases in 47 states in 2021 [1]. Evaluation of this data can reduce the impact of WNV and the progression to the life-threatening neuroinvasive disease seen in this patient. Personal protection in the form of insect repellent and long-sleeve clothing should also be encouraged.

## Conclusions

In this case report, we presented a patient with WNV, masked by suspected Guillain Barre syndrome. WNV-associated Guillain-Barré syndrome is an extremely rare sequela of WNV and may present with encephalitis, meningitis, or acute flaccid paralysis, as it did in this patient. Clinicians should maintain a high index of suspicion for WNV in patients with symptoms and lab findings consistent with GBS that present in the later summer months and reside in more tropical climates.
